# Brain gene expression profiles of *Cln1 *and *Cln5 *deficient mice unravels common molecular pathways underlying neuronal degeneration in NCL diseases

**DOI:** 10.1186/1471-2164-9-146

**Published:** 2008-03-28

**Authors:** Carina von Schantz, Juha Saharinen, Outi Kopra, Jonathan D Cooper, Massimiliano Gentile, Iiris Hovatta, Leena Peltonen, Anu Jalanko

**Affiliations:** 1 National Public Health Institute and FIMM, Institute for Molecular Medicine, Helsinki, Finland; 2 Genome Informatics Unit, University of Helsinki, Finland; 3 Folkhälsan Institute of Genetics, Helsinki, Finland; 4 Neuroscience Center, University of Helsinki, Finland; 5 Institute of Psychiatry, King's College, London; 6 University of Helsinki, Department of Medical Genetics and Research Program of Molecular Neurology, Biomedicum Helsinki, Finland; 7 University of Helsinki, Department of Medical Genetics and Research Programme of Molecular Medicine, Biomedicum Helsinki, Finland; 8 Wellcome Trust Sanger Institute, Wellcome Trust Genome Campus, Hinxton, Cambridge, CB10 1SA, UK

## Abstract

**Background:**

The neuronal ceroid lipofuscinoses (NCL) are a group of children's inherited neurodegenerative disorders, characterized by blindness, early dementia and pronounced cortical atrophy. The similar pathological and clinical profiles of the different forms of NCL suggest that common disease mechanisms may be involved. To explore the NCL-associated disease pathology and molecular pathways, we have previously produced targeted knock-out mice for *Cln1 *and *Cln5*. Both mouse-models replicate the NCL phenotype and neuropathology; the *Cln1-/- *model presents with early onset, severe neurodegenerative disease, whereas the *Cln5-/- *model produces a milder disease with a later onset.

**Results:**

Here we have performed quantitative gene expression profiling of the cortex from 1 and 4 month old *Cln1-/- *and *Cln5-/- mice*. Combined microarray datasets from both mouse models exposed a common affected pathway: genes regulating neuronal growth cone stabilization display similar aberrations in both models. We analyzed locus specific gene expression and showed regional clustering of *Cln1 *and three major genes of this pathway, further supporting a close functional relationship between the corresponding gene products; adenylate cyclase-associated protein 1 (Cap1), protein tyrosine phosphatase receptor type F (Ptprf) and protein tyrosine phosphatase 4a2 (Ptp4a2). The evidence from the gene expression data, indicating changes in the growth cone assembly, was substantiated by the immunofluorescence staining patterns of *Cln1-/- *and *Cln5-/- *cortical neurons. These primary neurons displayed abnormalities in cytoskeleton-associated proteins actin and β-tubulin as well as abnormal intracellular distribution of growth cone associated proteins GAP-43, synapsin and Rab3.

**Conclusion:**

Our data provide the first evidence for a common molecular pathogenesis behind neuronal degeneration in INCL and vLINCL. Since *CLN1 *and *CLN5 *code for proteins with distinct functional roles these data may have implications for other forms of NCLs as well.

## Background

The Neuronal Ceroid Lipofuscinoses (NCLs) are the most common group of inherited neurodegenerative diseases of children, with an estimated worldwide incidence of 1:12,500. Affected individuals suffer from visual impairment, seizures and dementia leading to early death [1]. The NCLs are a group of lysosomal storage disorders and display a characteristic accumulation of lysosomal autofluorescent lipopigments with a distinct ultrastructure in each form of NCL [2]. Targeted knock out mouse models of different forms of NCL also display broadly similar pathological findings: cortical atrophy, loss of GABAergic interneuron subpopulations, loss of thalamic relay neurons and pronounced gliosis [3-11]. However, the precise nature and timing of these events differs markedly between the different forms of NCL.

The NCLs are classified on the basis of the age of onset, clinicopathological features and genetic linkage, to ten different subtypes (CLN1-CLN10) and seven genes have so far been identified. Two very severe, early onset forms, CLN1 and CLN2 are caused by deficiencies of a lysosomal enzyme, palmitoyl protein thioesterase 1 (PPT1) and tripeptidylpeptidase 1 (TPP1) respectively [12,13]. The phenotypically milder forms CLN3, CLN5, CLN6 and CLN8, are caused by mutations in genes encoding proteins of unknown functions [14-20]. In addition, the deficiency of cathepsin D causes a congenital form of NCL, currently denoted as CLN10 [21]. The precise mechanisms by which mutations in these genes result in a dramatic neuronal degeneration are still unknown, but the broadly similar pathological and clinical profiles of the different NCLs suggest that common molecular pathways might link these diseases together, despite having a differing primary cause. Nevertheless solid evidence for molecular links between different forms of NCL is still scant. However, CLN5 is shown to interact both with the CLN3 and the CLN2 proteins [19].

To gain insights into the molecular pathogenesis of the various NCLs we have recently generated mouse models of the Infantile Neuronal Ceroid Lipofuscinosis (INCL, CLN1) and the Finnish variant form of Late Infantile Neuronal Ceroid Lipofuscinosis (vLINCL_Fin_, CLN5). The common pathological theme in both INCL and vLINCL_Fin _is the dramatic cortical atrophy, the origin of which has remained elusive in the human studies [1,2]. These *Cln1-/- *(*Ppt1*^*Δex4*^) and *Cln5*-/- mice replicate the appropriate clinicopathological presentation of human INCL and vLINCL_Fin_, respectively. *Cln1-/- *mice exhibit a severe, early onset disease, whereas *Cln5-/- *mice display a much milder NCL-like phenotype. Both of these models show characteristic ultrastructural findings, namely granular osmiophilic deposits (GRODs) in the *Cln1-/- *and curvilinear as well as fingerprint profiles in the *Cln5-/- *mice [5,6].

Here we have studied one month and four month old *Cln1-/- *(*Ppt1*^*Δex4*^) [5] and *Cln5 -/- *[6] mice to address the issue of a "shared common pathway" behind neurodegenerative changes in these two forms of NCL. In both mouse models global transcript profiling of the cortex revealed prominent alterations in genes involved in protein phosphorylation. Interestingly, the changes specific for *Cln1-/- *mice were associated with genes involved in neurogenesis and calcium homeostasis, whereas changes specific for *Cln5-/- *mice involved genes responsible for myelination and RNA processing. Comparing the data from both mice exposed 51 common genes, many of which were involved in a shared pathway affecting the neuronal growth cone – cytoskeletal dynamics. We utilized a custom-made tool to analyze locus specific gene expression and observed clustering of the key genes of this pathway, together with *Ppt1/Cln1*. Subsequent immunofluorescence analysis of cortical neurons further supported these findings, thus providing the first evidence for a shared molecular mechanism behind neuronal degeneration in the different forms of NCL.

## Methods

### Animals

Homozygous mutant *Cln1-/- *(Ppt1^Δex4^) mice [5] and *Cln5-/- *[6] mice were maintained on the same mixed C57BL/6Jx129SvEv strain background. All animals used in this study were a result of five generations of backcrossing onto C57BL/6J. We used systematically sampled brain tissue from 1 and 4 month old male mice for all experiments and wild-type littermate mice were used as controls for each mutant strain. The genotypes of the mice were determined by PCR of DNA from tail biopsies [5,6]. All animal experiments were performed in accordance with approved animal policies of the National Public Health Institute, Helsinki.

### Histological processing and immunohistochemistry

For histological analysis, 1 and 4 month old male *Cln5*-/-, *Cln1*-/- and age-matched control mice (*n *= 3/genotype) were sacrificed in a rising concentration of carbon dioxide, where after their brains were removed and bisected along the midline. One half of the bisected brain was frozen in liquid nitrogen and stored in -70°C and the other half was immersion fixed in 4% paraformaldehyde. Subsets of the brains were embedded in paraffin and 5 μm coronal sections where cut through the cerebrum and processed further as described previously [3]. Paraffin sections were immunohistochemically stained for β-tubulin III (Chemicon, 1:200) and F-actin (Alexa Fluor 488 Phalloidin, Molecular Probes) using a standard protocol [5].

### Quantitative tresholding image analysis

The expression of β-tubulin isoform III, and actin was measured by quantitative thresholding image analysis as previously described [3], with each marker analyzed blind with respect to genotype. 40 nonoverlapping images, on triplicate sections, were captured through each selected brain region and the optimal segmentation of immunoreactive profiles was determined using *Image Pro-Plus *image analysis software (Media Cybernetics) using a previously described semi-automated thresholding method based on the optical density of the immunoreactive product [3]. Macros were recorded to transfer the data to a spreadsheet for subsequent statistical analysis. Data were separately plotted graphically as the mean percentage area of immunoreactivity per field ± S.E.M. for each region.

### Gene expression profiling of the cortex

The remaining half of each bisected brain from 1-month-old and 4-month-old male *Cln1*-/-, *Cln5*-/- and age-matched control mice were used for gene expression profiling. To average out the inter-individual variability, RNA extracted from the entire cortical area of eight *Cln1-/- *mice, eight wild-type littermates, eight *Cln5-/- *mice and eight of their wild-type littermates, was pooled together into two wild-type and two knock-out samples per disease state. Thus, each pool contained RNA from four different mice and two pools were analyzed as replicates for each genotype. 5 μg of total RNA was labeled and fragmented according to the manufacturer's instructions (Affymetrix, Santa Clara, CA). Gene expression profiles were determined using the Affymetrix Mouse MOE 430A array (Affymetrix, Santa Clara, CA). Hybridization, post-hybridization washes, staining and scanning were performed as described previously [5]. Data normalization, filtering and determination of significance cut-offs were performed following rigorous standards, optimized to achieve a minimum of false positive discoveries [5]. Briefly, normalization was carried out using the Robust Multichip Average (RMA) model implemented in the R package *Affy*, using default settings. The normalized data was imported into the GeneSpring 6.0 data analysis software (Silicon Genetics, Redwood City, CA). Only probe sets scored as "present" by the Affymetrix MAS 5 algorithm in at least two of the four arrays passed the first filtering step, a second filter was applied to remove genes exhibiting inconsistent results across the two biological replicate samples representing each genotype. Expression values were averaged over the replicates and log-transformed to assure normally distributed values. Values differing at least two standard deviations from the mean were considered indicative a significant difference in expression.

### Gene Expression Pathway Analysis

In order to analyze the affected biological entities that may not have been defined by significant individual gene expression changes, a custom made non-parametric pathway analysis algorithm (related to [22]) was employed using the gene annotation data from the GeneOntology Consortium [23]. The Affymetrix "present" tagged probe sets were ranked by the median fold change values between wild type and *Cln1/5 *knock out mice to "upregulated" and "downregulated" vectors. The probe sets were then mapped to mouse genes using cross-references in Ensembl database and their associated GO annotations were collected. Our algorithm also utilized the directed topology of the GO-tree by traversing all available routes to the root of the GO tree and all encountered vertexes were added as GO annotations for the given gene. For detection of affected GO gene groups (here called "pathways"), an iterative cumulative hypergeometric distribution p-value based calculation was used, where the objective is to find the optimum p-value for a set of genes which belong to the same annotated GO gene collection (maximal regulation for the pathway). The algorithm works by traversing through the ranked list of genes and at every occurrence of a gene belonging to the given pathway, the hypergeometric p-value is calculated, answering the question "how likely is it to see this many genes (*k*) that belong to the studied pathway this high-up in the ranked list of genes (*j)*, when there are altogether *t *genes that belong to the pathway amongst *n *genes in the experiment" (see (1)).

$$p(j,k,t,n)=1-\sum_{c=0}^{k-1}\frac{\left(\begin{array}{c} j\\ c \end{array}\right)\left(\begin{array}{c} n-j\\ t-c \end{array}\right)}{\left(\begin{array}{c} n\\ t \end{array}\right)}$$

Further, exhaustive permutations by randomizing the gene ranks was done for each GO category, followed by an interpretation of an empirical p-value from the distribution of 10,000 permutation cycles.

### Locus Specific Gene Expression Analysis

The locus specific gene expression data analysis was done by first mapping the Affymetrix probe sets to their corresponding genes in the mouse genome in Ensembl database version 43. The Affymetrix "present" tagged probe sets were then ranked as "upregulated" and "downregulated" between wild type and *Cln1/5 *knock out mice by calculating median fold changes. The probesets for *Cln1 *and *Cln5 *were removed from the analyses of *Cln1-/- *and *Cln5-/- *mice, respectively. Next the gene expression at each locus of the mouse genome was systematically studied. Briefly, by starting at each gene along each mouse chromosome, each referred mouse gene was used as a starting point for a locus. The length of a given locus was systematically extended, ranging between 5,000 to 20,000,000 base pairs and 2 to 100 genes. Each generated locus containing *t *genes within those limits was analyzed for regulation of expression by a similar iterative hypergeometric distribution as with Gene Expression Pathway Analysis. The *t *i.e. how many genes belong to the locus, was optimized as the number providing the strongest regulation for the given locus (that is, the regulation of expression of the locus was represented by a length of the locus that was within the given locus size boundaries and provided the strongest regulation). This search was repeated using all the mouse genes as locus starting points and an optimal regulation for these was calculated. Further, in addition to the nominal p-values, empirical p-values were calculated for all studied loci sizes (2...100 genes) each with 10,000 permutations using randomized gene/probeset ranked lists.

### Neuronal cell culture and immunofluorescence staining

E15.5 primary cortical neuron cultures were prepared from *Cln1-/-*, *Cln5 -/- *and wild type control mouse embryos as described previously [24]. For immunofluorescence staining, the neurons were grown on coverslips for eight days and were then fixed with 4% paraformaldehyde in PBS, permeabilized and immunostained as described previously [25]. The primary antibodies used were anti Synapsin 1 and 2 rabbit antiserum and mouse monoclonal anti Rab 3 (Synaptic Systems GmbH, 1:100 and 1:1000 respectively), Gap43 (1:1000) and mouse monoclonal anti β-tubulin, isoform III (Chemicon, 1:200). Alexa Fluor 488 Phalloidin (Molecular probes, as recommended by the manufacturer) was used for visualizing F-actin. The coverslips were mounted in GelMount (Biomeda Corp., Foster City, CA) and viewed with a Leica SP confocal microscope. The analyses were performed from three different preparative batches of neurons with duplicate immunostainings from each batch.

### Mouse brain membrane preparation and Western blotting

Flash frozen brains were homogenized in a buffer (2.5 mM KCl, 250 mM sucrose, 25 mM HEPES pH 7.4, protease, and 2 mM DTT) and separated into membrane and cytoplasmic fractions as described previously [26]. The membrane and cytoplasmic fractions were electrophoresed on 14% SDS-PAGE gels and transferred onto nitrocellulose membranes by electroblotting. The immunostaining was performed with primary antibodies for Synapsin 1, synapsin 1+2 rabbit antiserum (1:1000) and mouse monoclonal anti Rab 3 (1:100), Gap43 (1:1000) and mouse monoclonal anti β-tubulin, isoform III (1:200). HPR-conjugated secondary antibodies were used diluted to 1:5000. Western blotting was performed as recommended by the manufacturer (Amersham Biosciences). The analyses were repeated twice.

## Results

### Gene expression profiling of the cortex reveals differential expression of genes with known functions in neuronal signaling and structure

Human as well as other vertebrate NCL diseases are typified by local astrocytic activation and a gross upregulation of inflammation related genes in the later stages of disease progression [5,6]. Here we have addressed the global changes in transcript profiles in the cerebral cortex of the *Cln1-/- *and *Cln5-/- *mice with the hypothesis that this would provide us with clues of critical metabolic pathways that contribute to the neurological phenotype and neuronal degeneration.

#### The Cln1 -/- mouse

The combined analysis of the replicate microarray datasets from the cortex of 1 month old *Cln1*-/- mice revealed a total of 417 differentially expressed genes compared to controls, of which 261 were upregulated and 156 downregulated (**see **Additional file 1, see methods). None of the upregulated genes exceeded a fold change of 2, the most upregulated gene being cytotoxic granule-associated RNA binding protein 1 (*Tia1*, fold change of 1.6). The most downregulated gene was *Cln1 *(*Ppt1 *with a fold change of -3.5) followed by protein tyrosine phosphatase receptor type F (*Ptprf*, fold-change of -2.2).

Similar analysis of 4 month old *Cln1-/- *and control mice revealed 164 differentially expressed genes, of which 137 were upregulated and only 27 downregulated (**see **Additional file 1). The most upregulated gene was adenylate cyclase-associated protein 1 (*Cap1*) being over 32-fold upregulated, followed by glial fibrillary acidic protein (*Gfap*), which was 16.6-fold upregulated. Most downergulated genes included inactive X specific transcripts (*Xist*), G protein-coupled receptor, family C, group 5, member B (*Gprc5b*) and *Ptprf *(with fold changes of -7.0, -3.7 and -3.3 respectively).

#### The Cln5 -/- mouse

Analysis of the datasets of 1 month old *Cln5-/- *mice revealed 283 differentially expressed genes compared to controls, with 76 up- and 206 downregulated genes. In the cortex of *Cln5-/- *mice, the most upregulated gene was *Cap1 *(3.9-fold increased), the same gene that was identified as the most upregulated in 4 month old *Cln1*-/- mice (**see Additional **1). The most downregulated gene was chemokine (C-C motif) ligand 21a (*Ccl21a*), with a fold change of – 1.7.

At the age of 4 months, 114 differentially expressed genes were revealed in *Cln5-/- *mice, of which 34 were upregulated and 80 downregulated. The most prominent change was the -6.4 fold downregulation of kinesin family member 5C (*Kif5c*), followed by DEAD (Asp-Glu-Ala-Asp) box polypeptide 6 (*Ddx6*) and *Gprc5b *(-6.0 and -5.7 respectively). The most upregulated gene was dentatorubral-pallidoluysian atrophy protein (*Drpla*) with a fold change of 4.1 (**see **Additional file 1). The results of the gene expression profiling of both *Cln1-/- *and *Cln5-/- *datasets were validated by quantitative real-time PCR analysis of selected genes (data not shown).

### Combined analyses reveal that neuronal growth cone and axon guidance pathways are differentially expressed in both CLN1 and CLN5

#### Single gene analysis

**Figure 1:**
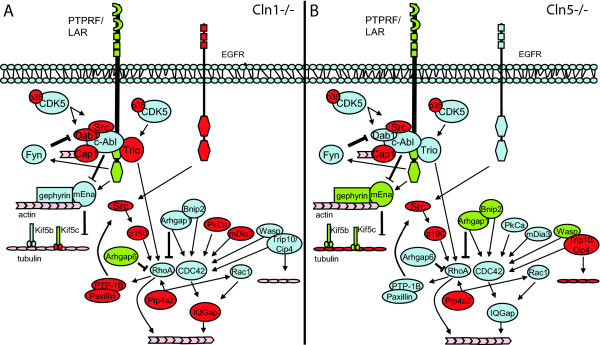
**Common affected pathways in *Cln1 *and *Cln5 *deficient mouse models.** A schematic picture of protein-protein interactions between genes that are differentially expressed in both mouse-models. Upregulated genes are shown in red and downregulated genes in green. Genes with no changes in expression are shown in blue. Genes with changes only in one replicate experiment are shown in pink.

To address the "common molecular pathways" affected in both mouse models, we aimed to identify sets of genes that showed similar patterns of changes in gene expression in both *Cln1-/- *and *Cln5-/- *mice. Comparison of the microarray data sets revealed 51 genes that were differentially expressed in both models (Table 1). Among these we could further pinpoint genes that encode for proteins critical for the neuronal growth cone – cytoskeletal dynamics, including *Ptprf*, rous sarcoma oncogene (*Src*), *Cap1*, protein tyrosine phosphatase 4a2 (*Ptp4a2*), *Kif5c*, contactin associated protein 1 (*Cntnap1, p190*) and the Cdk5 regulatory subunit p35 (*Cdk5r1*). These proteins may further link the growth cone and cytoskeletal dynamics to axon guidance/growth via the ras homolog gene family, member A (*RhoA*), cell division cycle 42 homolog (*Cdc42*) and RAS-related C3 botulinum substrate 1 (*Rac1*). The protein products of the differentially expressed genes in the common affected pathway are shown in Fig 1.

**Table 1:** Differentially expressed genes in both *Cln1-/- *and *Cln5-/- *mice at 1 and 4 months of age. Double/triple values indicate multiple appearances in the gene expression data.

**ID number**	**Cln1 1 mo**	**Cln1 4mo**	**Cln5 1mo**	**Cln5 4mo**	**Symbol**
NM_011213	**-2,2/-1,8**	**-3,3/-1,9**	**-1,3**	**2,6/2,5**	Ptprf
BE134116	**-1,5**	**3,8**	**1,7**	**-3,7**	Ptp4a2
AF378831	**-1,2**	**-3,7**	**1,6**	**-5,7**	Gprc5b/raig2
BC023103	**1,5/1,3**	**2,7**		**4,0**	Camkk2
AI844677	**-1,5**	**2,1**		**-6,4**	Kif5c
BC021452	**-1,2**	**2,2**		**-3,2/-6,0**	Ddx6
BG071068	**1,3**	**-2,4**		**-5,0/-3,4**	Gnb1
BB647052	**1,3**	**1,8**	**1,5**		Tex261
BG065186	**-1,2/-1,3**	**2,3**	**1,4**		Ywhaz
BM214109		**2,6**	**-1,3**	**-2,5**	Tpr
BI692255	**-1,5**		**-1,3**	**-3,8**	Prkwnk1
BE447456	**-1,2**		**-1,3/-1,5**	**-2,7**	Khdrbs1/sam68
AI788623	**1,3**		**-1,4**	**-3,6/-2,7**	Ramp2
AY026947	**-1,3**		**-1,3**	**-2,6**	Rab3c
AU080702	**1,3**			**2,8**	Mapt
BG962849	**1,5**			**2,7**	Foxp1
AK002933	**1,2**			**2,6**	Nrgn
AU067745	**1,2**			**2,6**	Elavl3
BE952057	**1,2**			**2,5**	Fscn1
U00689	**1,6/1,3**			**2,5**	Tia1
AV139249	**1,3**			**2,4**	Bsn
BB419318	**-1,3**			**-3,5**	Gda
BB283759	**-1,3**			**-3,5**	Csnk2a1
AV083741	**-1,3**			**-3,4**	Mtdh
NM_018733	**-1,2**			**-3,0**	Scn1a
NM_008066	**-1,3**			**-2,7**	Gabra2
AW060288	**-1,2**			**-2,6**	Qk
NM_008845	**-1,2**			**-2,5**	Pip5k2a
BG067003	**-1,2**			**-2,4**	Zrf2
BC005446		**32,2**	**3,9/3,2**		Cap1
NM_018798		**2,4**	**1,3**		Ubqln2
BM224327		**2,4**	**1,3**		Fcgr2b
BB538325		**2,2**	**-1,4**		Ccnd1
AV102733		**1,8**	**-1,3**		Scpep1
AW049660		**-1,9/-1,8**	**-1,3**		Nfix
AV147875		**2,3/2,7/2,9**		**-3,1**	Vim
AF363030		**2,9**		**3,2**	Ogt
AJ457190		**2,3**		**3,4**	Phospho1
NM_007881		**2,1**		**4,1**	Drpla
NM_016782		**2,1**		**3,3**	Cntnap1
BG868120		**2,0**		**2,4**	Src
NM_016789	**1,2**		**1,7**		Nptx2
AA673371	**1,4/1,3/1,2**		**1,4**		4933439C20Rik
NM_010444	**1,2**		**1,3**		Nr4a1
AK019164	**-1,2**		**-1,5**		Mpdz
BC006680	**-1,2**		**-1,4/-1,3**		Ubc
NM_016916	**-1,2**		**-1,3**		Blcap
AF370126	**-1,3**		**-1,3**		Dner
NM_011599	**-1,5**		**-1,3**		Tle1
BB429612	**1,2**		**-1,3**		Scn8a
BQ175532	**-1,2**		**-1,3/-1,4**		Ahi1

#### Functional classes

**Figure 2:**
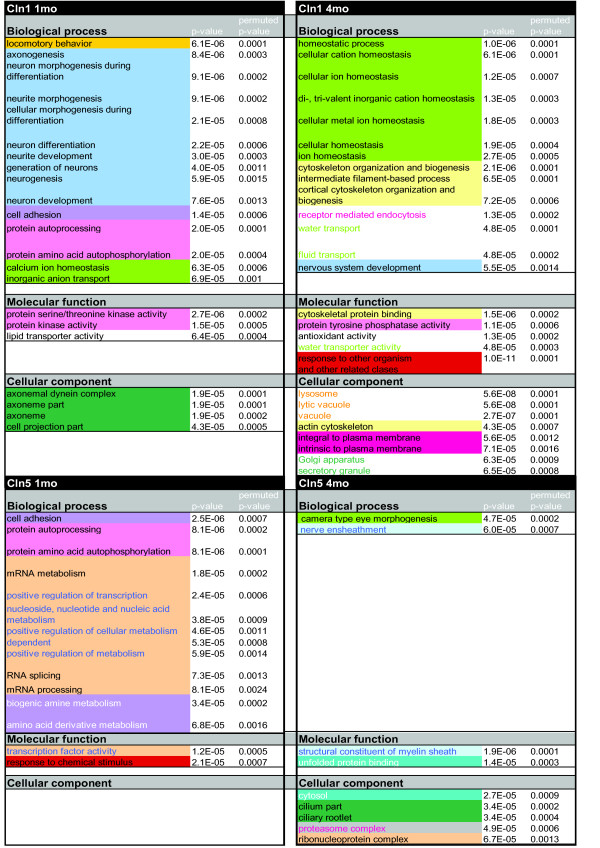
**Gene expression differences according to gene ontology classes in: *Cln1-/- *1mo array, *Cln1-/- *4mo array, *Cln5-/- *1mo array, *Cln5-/- *4mo array.** All the significant categories are shown.

In order to analyze the affected biological pathways, which may not be defined by significant individual gene expression changes, a custom made non-parametric pathway analysis algorithm was employed. When analyzing the differentially expressed genes of the 1 month old *Cln1-/- *mice we found several functional classes to be significantly regulated. The most pronounced alterations were seen in genes related to development of neuronal entities, followed by signaling pathways related to phosphorylation and calcium ion homeostasis (Fig 2). When analyzing the 4 month old *Cln1-/- *mice we discovered upregulation of inflammation associated pathways. In addition, we found that *Calcium homeostasis, Cytoskeleton *and *Lysosomes *represented the most significantly affected categories (Fig 2).

Our analysis of the 1 month old *Cln5-/- *mice revealed regulation of cell adhesion, RNA processing and transcription, as well as phosphorylation related pathways (Fig 2). In the 4 month old *Cln5-/- *dataset our analysis pinpointed regulation in nerve ensheatment/myelination as well as in and camera eye development (Fig 2).

Collectively, the transcript profiling of both *Cln1*-/- and *Cln5 -/- *mice highlighted changes in genes involved in phosphorylation. Additionally, changes in neurogenesis and calcium homeostasis associated genes were more pronounced in young *Cln1-/- *mice whereas changes in the expression of genes associated with myelination and RNA processing characterized the *Cln5*-/- mice.

### Co-regulation of multiple genes in the *Cln1 *genetic locus

**Figure 3:**
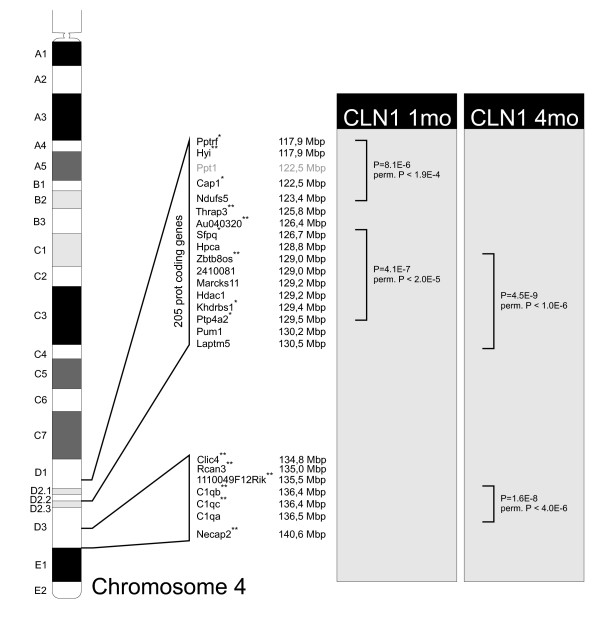
**Chromosomal location of coregulated genes.** Several differentially expressed genes show significant clustering in around *Ppt1 *on mouse chromosome 4, and might therefore be coregulated. The probesets for Ppt1 were excluded from the dataset and thus Ppt1 is shown in gray. The genes participating in the regulated loci or being independently regulated are shown, together with their location in base pairs in mouse chromosome 4. Genes that exhibit expression differences in both mouse models are indicated with *. Expression changes only in the *Cln1-/- *mouse are indicated with **.

Interestingly, three key players of the identified neuronal growth cone/axon guidance pathway are located in close proximity of the *Cln1 *gene in the mouse genome, potentially indicating a close functional relationship or even interdependence between these gene products. As the same set of genes is differentially expressed also in the *Cln5-/- *mouse, we ruled out the possibility of this being due to knocking out the *Cln1 *gene and therefore affecting possible regulatory regions of these other genes. *Ptprf*, *Ptp4a2 *and *Cap 1 *(Table 1) are located in the vicinity of the *Cln1 *gene on mouse chromosome 4 (Fig 3), with a similar clustering of these four genes on the human chromosome 1q32. To assess the relevance of this finding, we used an in-house developed data analysis tool for locus specific gene expression. We searched for coordinated regulation of gene expression at each locus of the mouse genome, with size limits ranging from 5000 to 20,000,000 base pairs. This analysis revealed that the most significantly affected locus in the *Cln1-/- *mouse datasets was 4D2.2 in both 1 month and 4 month old mice. The most significant area on 4 D2.2 in the 1 month *Cln1 *dataset was flanked by Ptp4a2 and splicing factor proline/glutamine rich (Sfpq) (nominal p = -4.1 × 10^-7^; permuted p = 2.0 × 10^-5^). Other highly significant clusters were found in the 4D1 area, surrounding the Ppt1 locus, ranging from Ptprf to NADH dehydrogenase (ubiquinone) Fe-S protein 5 (Ndufs5) (nominal p = -8.1 × 10^-6^; permuted p = 1.9 × 10^-4^). (Fig 3). The most significant area on 4 D2.2 in the 4 month *Cln1 *dataset was flanked by lysosomal-associated protein transmembrane 5 (Laptm5) and zinc finger and BTB domain containing 8 opposite (Zbtb8os) (nominal p = -4.5 × 10^-9^; permuted p = 1.0 × 10^-6^). (Fig. 3). Another significant locus was found on 4 D3. This locus included three complement component genes, namely complement component 1, q subcomponent, chains B, C and A (C1qb, C1qc and C1qa) (p = 1.6 × 10^-8^, permuted p = 4.0 × 10^-6^) (Fig. 3). No significant clustering in these specific regions were found in the *Cln5-/- *mouse dataset, although many of the genes located in the same region were also differentially expressed in *Cln5-/- *mice.

Currently, the functional significance of such gene clustering is unknown, but emerging data from other gene clusters of the genome could imply that genes within evolutionary preserved linkage groups retain their clustering since they code for proteins with critical functional interactions. Since *Ptprf, Cap1 *and *Ptp4a2*, the genes found in the clusters around *Ppt1*, are connected to the same signaling pathway (Fig 1), and since they are differentially expressed in both disease models, this may imply a distinct functional relationship between these genes.

Taken together, a common affected signaling pathway between *Cln1-/- *and *Cln5-/- *mice was revealed by a combined analysis of transcript profiles (Fig 1). This pathway is connected to phosphorylation and further affects the cytoskeleton and growth cones in neurons. Additionally, genes for the three key players of this pathway were shown to be clustered on the same chromosomal region with the *Ppt1 *gene, potentially indicating a close functional relationship or even interdependence between these gene products.

### Altered subcellular localization of proteins implicated in cytoskeletal dynamics and growth cone anatomy

Our gene expression profiling data with subsequent analysis of the affected biological pathways as well as utilization of the locus-specific gene expression tool suggested a potential impairment of protein phosphorylation genes related to cytoskeleton-mediated growth cone anatomy in *Cln1 *and *Cln5 *deficient mice. We assessed possible changes in protein tyrosine phosphorylation between knock out and wild-type control mice in western blot analyses of CNS tissue but could not identify statistically significant differences (data not shown).

**Figure 4:**
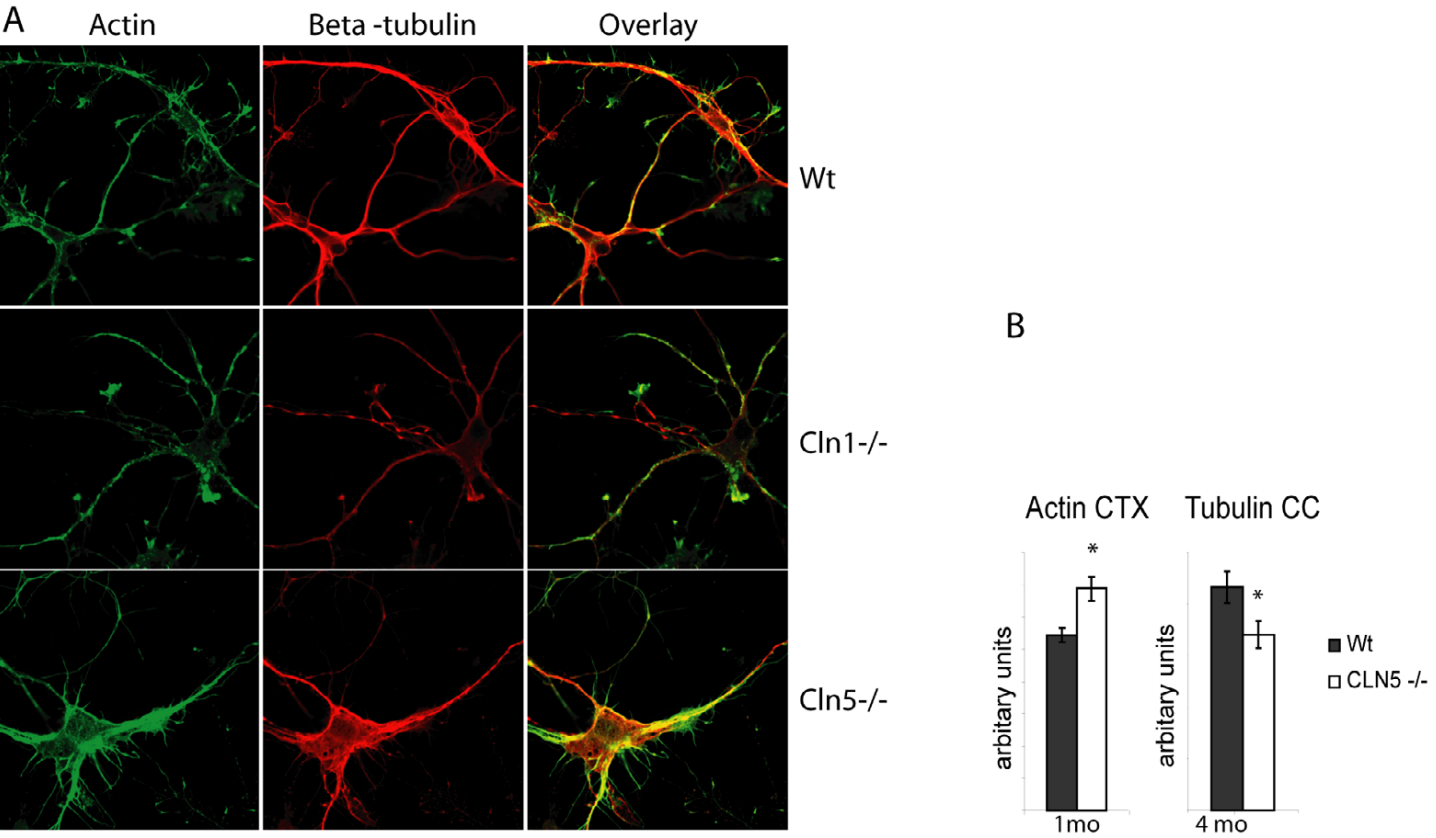
**A) Immunofluorescence analysis of actin and β-tubulin staining in E15 primary cortical neurons of wt, *Cln1-/- *and *Cln5-/- *mice.** Actin is visualized in green and β-tubulin in red. The overlay shows the colocalization of the two proteins in yellow. **B) **Quantitative threshold imaging of actin and β-tubulin staining in the *Cln5-/- *mouse brain. Significant p-values, with p < 0.05 are indicated with *. **Ctx**, cortex and **cc**, corpus callosum.

To assess the putative defects in growth cone anatomy, we first analyzed potential differences in the intracellular localization or quantity of actin and tubulin, two generally utilized markers for initial characterization of the integrity of neuronal projections. We performed double immunofluorescence analysis of E15 primary cortical neurons of wild type as well as *Cln1 *and *Cln5 *deficient mice. Neurons from *Cln1-/- *mice displayed pronounced actin staining in growth cones, but a relative lack of actin and β-tubulin immunostaining in the cell soma, as compared to controls (Fig 4), whereas neurons from *Cln5-/- *mice exhibited particularly intense actin staining in the neuronal soma and processes (Fig 4). Consistent with these data, thresholding image analysis of immunohistochemically stained brain sections revealed prominent changes in actin immunostaining in the cortex of 1 and 4 month old *Cln5-/- *mice (Fig 4). Abnormal intracellular localization of β-tubulin was not evident in stained cell-cultures from *Cln5-/- *mice, but thresholding analysis of brain sections revealed a significant reduction in the β-tubulin staining in the cortex of 4 month old *Cln5-/- *animals (Fig 4). Western blot analysis of soluble and membrane-bound intracellular fractions from four months old mice further demonstrated reduced levels of β-tubulin in the cytoplasmic fraction of *Cln5-/- *mouse brains. In the case of β-tubulin, the changes in gene expression profile, indicating upregulation at 1 month old and no significant change at 4 month *Cln1-/- *mouse cortex, did not translate into the protein level.

**Figure 5:**
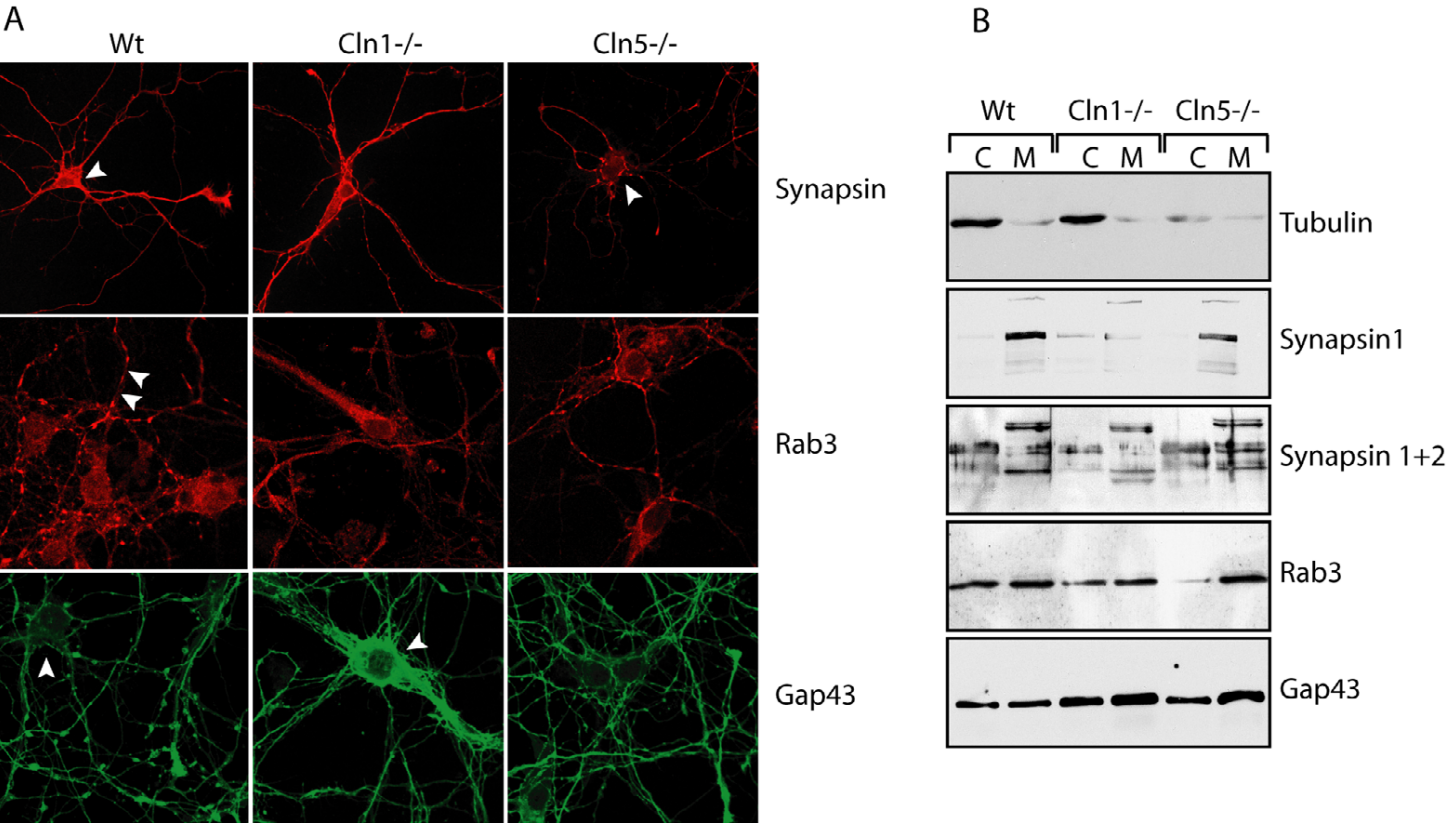
**A) Immunofluorescence analysis of Synapsin, Rab3 and GAP-43 staining in E15 primary cortical neurons of wt, *Cln1-/- *and *Cln5-/- *mice.B) **Western blot analysis of cytoplasmic and membrane bound fractions of cytoskeletal, growth-cone and synapse assembly proteins. Antibodies: β-tubulin, synapsin 1, synapsin 1&2, Rab3 and Gap43.

To assess the potential aberrations in the intracellular staining of growth-cone related proteins, we also immunostained neuronal cultures from *Cln1 *and *Cln5 *deficient mice for the growth cone assembly protein GAP-43 and synapsin, two well known markers for growth cone integrity, together with Rab3 which was differentially expressed according to our transcript profiling data. These analyses revealed that GAP-43 positive axonal varicosities were remarkably sparse especially in the *Cln1-/- *neurons; although intense membrane associated GAP-43 immunoreactivity was evident. This finding was confirmed by western blot analysis, which revealed a more prominent localization of GAP-43 in membrane-bound fractions in both *Cln1-/- *and *Cln5-/- *mouse brains (Fig 5). Synapsin immunostaining of *Cln5*-/- neurons revealed abnormal membrane binding around the cell soma whereas the *Cln1*-/- neurons showed abnormally faint Rab3 immunoreactivity, especially within neurites (Fig 5). These data could indicate reduced amounts of these proteins within cultured neurons, or alternatively may be a sign of their mislocalization. Western blot analysis of soluble and membrane bound fractions of brain tissue revealed further changes in the processing and localization of synapsins in *Cln1-/- *mice, but only negligible changes in *Cln5-/- *mice. In contrast, Rab3 was almost completely absent from the cytoplasmic fraction of *Cln5*-/- mice, but appeared unchanged in *Cln1-/- *brains (Fig 5). These results demonstrating altered subcellular distribution of cytoskeleton and growth-cone related proteins in both *Cln1-/- *and *Cln5-/- *neurons as well as brain tissue, provide direct evidence for a functional significance of the abnormalities observed in transcript profiles and further support the observation that cytoskeletal and growth cone mediated pathways are disturbed in INCL and vLINCL.

## Discussion

In this report, we have explored the hypothesis that a common molecular pathway may underlie some of the aspects of neurodegeneration the characteristic feature of neuronal ceroid lipofuscinoses. The developments of mouse models for NCLs as well as the advancements of microarray technologies have opened the way for discovering affected molecular pathways on a genome-wide scale. By using two mouse models of NCL; the *Cln1-/- *(*Ppt1*^*Δex4*^) [5] and the *Cln5-/- *[6] mice, we investigated if any evidence could be collected for shared molecular pathways. At autopsy of NCL patients the cortex is the most severely affected brain region, and hence we decided to perform global transcript profiling of *Cln1-/- *and *Cln5-/- *cortices. Previous data obtained from gene expression profiling of *Cln1-/- *mouse cortex tissue has provided information on the importance of neuroinflammation in the late stage of the disease [5]. The transcript profiling of *Cln5*-/- cortices has implicated inflammation and defects in myelination at the ages of 3 and 4.5 months [6]. Comparisons between previous analyses of NCL mouse models suggested marked upregulation of inflammation and neurodegeneration related genes in *Cln1-/- *and *Cln3-/- *mice whereas the changes in the *Cln5-/- *mice were less prominent at the level of individual gene expression [27,28]. However, differences in the methods used made the direct comparison between mouse models problematic. Here we utilized simultaneous sample preparation, rigorous microarray data filtration and a custom made pathway analysis algorithm and were able to identify a common affected biological pathway between the *Cln1-/- *and *Cln5-/- *mouse models.

Currently the initial cascade of events leading to glial activation and neurodegeneration are unknown [5,6,29] and therefore a global transcript profiling may provide an insight into early events in the disease progression. The comparison of two microarray data sets provides a means to determine how changes in gene expression relate to specific disease mechanisms. We identified a set of differentially expressed genes which were connected to same functional pathway in these two mouse models, and were also able to detect some "model specific" distinct differences between the cortical expression profiles. These features, potentially specific to each disease, included significant changes in genes involved in neurogenesis and calcium homeostasis (*Cln1-/- *mice) as well as genes involved in myelination and transcription (*Cln5-/- *mice).

Recent analyses showed that neuronal differentiation was not dramatically altered in the *Cln1-/- *mice but alterations in calcium homeostasis were evident in two different *Cln1 *deficient mouse models [30,31]. Myelination related changes were observed in our previous gene expression profiling analyses of 3 and 4.5 month old *Cln5-/- *mice [6] and were replicated here in the 4 month old *Cln5-/- *mice. This finding is not unexpected since the loss of myelin is a well-known feature in vLINCL patients [32,33]. Differential expression of genes relating to eye-development is interesting and expected, since blindness is the first symptom that can be observed in *Cln5-/*- mice [6]. However, the most significant finding specific to *Cln5-/- *mice was the differential expression of genes related to RNA processing and transcription. It is currently not known how this finding could relate to vLINCL, and further studies, specifically directed to systematic screening of RNA processing and transcription will be needed.

Our data imply that changes in phosphorylation related genes are common to both INCL and vLINCL. Interestingly, three phosphorylation and cytoskeleton related genes, differentially expressed in both mouse models, cluster around *Cln1 *on mouse chr 4. In the present study we have therefore developed a bioinformatic tool demonstrating the statistical significance of this finding. Genes that are part of the same metabolic pathway may be clustered in the same chromosomal region. Such linkage groups or gene clusters are not necessarily compact, but may correspond to comparatively large regions with high concentrations of functionally related or interdependent genes [34]. We propose that the observed regional clustering of these genes provides further evidence for their functional interactions and their link with *Cln1 *pathogenesis. Even more importantly, the dysregulation of the same genes in the *Cln5-/- *mouse cortex might implicate a link between the two forms of NCL. Further studies with systematic sampling and global analyses of all the available mouse models for NCLs (reviewed in [29]) should provide information on whether the differential expression of this gene cluster represents a hallmark of NCL diseases in general.

Several protein tyrosine kinases (PTKs) and protein tyrosine phosphatases (PTPs) showed significantly changed expressions. One of the functions that PTKs and PTPs regulate is growth cone motility, axon outgrowth and guidance [35]. *Ppt1*/*Cln1 *has been localized to axonal presynaptic vesicles and is also present in neuronal growth cones [24,36], suggesting a role for this soluble enzyme in these cellular compartments. Indeed, our immunofluorescence data suggest that the subcellular distribution of the growth cone specific GAP-43 protein is perturbed in *Cln1 *deficient cortical neurons. This feature is shared by *Cln5 *deficient neurons and suggests that a defect in growth cone anatomy may be common to these two forms of NCL.

Our transcript profiling also revealed a downstream impact of *Cln1 *and *Cln5 *deficiency upon the cytoskeleton. This was substantiated by cytoskeletal aberrations in the pathway analysis of the *Cln1-/- *mice and by significantly changed expression levels of individual actin and microtubulus-related genes in the *Cln5-/- *mice. This influence extended to a redistribution of actin that was most pronounced in the *Cln5-/- *mice. These data are consistent with recent observations in a mouse model of juvenile NCL, caused by the deficiency of CLN3 transmembrane protein, which is also linked to cytoskeletal abnormalities and intracellular trafficking defects [37,38]. In this respect it will be important to determine the impact of cytoskeletal rearrangements in *Cln1-/- *and *Cln5-/- *mice and whether these result in similar intracellular trafficking defects.

## Conclusion

We verified our findings from the transcript profiles with various methods to establish some functional support for a "common pathway" in these two forms of NCL. A common signaling pathway connected to phosphorylation and further affecting the cytoskeleton and growth cones in neurons was revealed to be affected in both *Cln1-/- *and *Cln5-/- *mice. Several genes coding for proteins in this pathway were shown to be clustered on the same chromosomal region with the *Ppt1 *gene. These fndings provide a possible mechanistic link between INCL and vLINCL diseases. Indeed, because the role of the functionally uncharacterized Cln5 protein remains unclear these studies may offer clues to the unraveling of its possible function(s). Previous studies have concentrated on biochemical approaches to find interaction partners for proteins and to reveal the disease processes. We have here shown that exposing affected metabolic pathways can provide an alternative route to approach the functional roles of proteins affected in genetic diseases.

## Authors' contributions

CvS carried out all of the experimental work, participated in the analysis of the microarray results and drafted the manuscript. OK participated in the study design concerning neuronal cell cultures and immunofluorescence stainings. JDC participated in the study design concerning the quantitative tresholding image analysis. MG carried out the gene expression analysis. IH participated in the microarray study design and drafting of the manuscript. JS created the analysis program for the locus specific gene expression analysis and participated in the gene expression pathway analysis. LP participated in the study design. AJ conceived of the study, participated in its design, coordination and drafting of the manuscript. All authors read and approved the final manuscript.

## Supplementary Material

Additional file 1 The differentially expressed genes in the Cln1-/- and Cln5-/- mice compared to wildtypes. A) The 261 upregulated genes in the Cln1 1mo model. B) The 156 downregulated genes in the Cln1 1 mo model. C) The 137 upregulated genes in the Cln1 4 mo model. D) The 27 downregulated genes in the Cln1 4 mo model. E) The 76 upregulated genes in the Cln5 1mo model. F) The 206 downregulated genes in the Cln5 1 mo model. G) The 34 upregulated genes in the Cln5 4 mo model. H) The 80 downregulated genes in the Cln5 4 mo model. Click here for file
